# Complete genome of a *Pseudomonas* sp. KK18 isolated from an Antarctic sediment exhibited genes related to plant growth-promoting potential

**DOI:** 10.1128/mra.00163-25

**Published:** 2025-06-09

**Authors:** So-Yeon Eom, Byeollee Kim, So-Ra Han, Sang-Hee Jung, Yuri Kim, Tae-Jin Oh

**Affiliations:** 1 Department of Life Science and Biochemical Engineering, Graduate School SunMoon University 35022 , Asan, South Korea; 2 Genome-based BioIT Convergence Institute, Asan, South Korea; 3 Department of Dental Hygiene, Gangneung Yeongdong University, Gangneung, South Korea; 4 Department of Pharmaceutical Engineering and Biotechnology, SunMoon University 35022 , Asan, South Korea; The University of Arizona, Tucson, Arizona, USA

**Keywords:** Antarctic Ross Sea, sediment, *Pseudomonas *sp, complete genome, plant growth-promoting bacteria

## Abstract

Here, we report the complete genome sequence of *Pseudomonas* sp. KK18, isolated from the Antarctic Ross Sea sediment. The isolate has a genome size of 7.3 Mb, 61% GC content, 100% completeness, and 0.36% contamination. The genome harbors genes associated with plant growth promotion and the biosynthesis of secondary metabolites.

## ANNOUNCEMENT

Plant growth-promoting bacteria (PGPB) contribute to soil health and plant resilience through mechanisms such as nutrient solubilization and siderophore production (1). *Pseudomonas* spp. are well-known PGPB with beneficial traits for plant-microbe interactions (2). Strains from extreme Antarctic environments exhibit plant growth-promoting activities, highlighting their potential as cold-active biofertilizers (3).

*Pseudomonas* sp. KK18 (KK18) was isolated from Ross Sea sediment collected during a Korea Polar Research Institute expedition. For bacterial isolation, 0.5 g of wet sediment was suspended in 2 mL of 0.9% saline solution and incubated at 55°C in a water bath for 6 min. Serial dilutions (10⁰–10⁻³) were incubated on R2A agar (MB Cell, Korea) at 15°C for 30 days. To obtain a single colony for contamination check, the strain was re-streaked on the R2A agar (MB Cell, Korea) plate. A liquid culture from the isolated colony was then used for 16S rRNA gene amplification with universal primers 27F and 1492R. Genomic DNA was extracted using the DNeasy Blood & Tissue Kit (Qiagen, Netherlands) following the manufacturer’s protocol. 16S rRNA was sequenced was sequenced via Sanger sequencing (Cosmo Genetech, Korea). Taxonomic identity was assigned using the EzBioCloud database (4). The isolated bacterial strains were preserved in a 20% (vol/vol) glycerol suspension at −70°C for storage.

The same genomic DNA extracted using the method above was used for both Illumina and Oxford Nanopore Technologies (ONT) sequencing. For Illumina sequencing, libraries were prepared using the TruSeq DNA Nano Kit (Illumina, USA) without size selection, and sequencing was conducted on a NovaSeq 6000 platform (2 × 150 bp paired-end reads) following standard protocols. For ONT sequencing, libraries were prepared using the 1D Ligation Sequencing Kit (SQK-LSK110) and the Native Barcoding Expansion Kit (EXP-NBD104), without size selection. Sequencing was performed using GridION with FLO-MIN106 (R9.4) flow cells and high-accuracy basecalling. ONT sequencing generated 41,843 reads (345.8 Mb, N50: 8,351 bp), and Illumina yielded 17,164,028 reads (2.6 Gb), with coverages of 133× (ONT) and 357× (Illumina).

Following Wick et al. (2023), we assembled a high-quality genome by performing long-read assembly, followed by long-read and short-read polishing (5). Genome assembly was performed using Trycycler (v0.5.4) (6), Flye (v2.9.2) (7), Medaka (v1.12.1) (8), Polypolish (v0.6.0) (9), and POLCA (v0.3.1) (10). Circularization was confirmed by Trycycler in reconciling contig steps (11). Genome quality was assessed using QUAST (v5.2.0) (12) and CheckM (v1.2.2) (13). Taxonomic classification was based on ANI calculated using OrthoANI  (14). Annotation was performed with Prokka (v1.14.6) (15), eggnog-mapper (v2.1.9) (16), and RAST (17). For all software, default parameters were used unless otherwise specified.

The genome characteristics of KK18 are presented in Table 1. The complete genome of KK18 consists of a single circular chromosome of 7,253,342 bp with a G + C content of 61.0%. Genome annotation revealed 6,461 protein-coding genes, 66 tRNAs, and complete rRNA operons (5S, 16S, and 23S). Average nucleotide identity (ANI) analysis showed the highest similarity (98.74%) to *Pseudomonas* sp. NC02. Building on the presence of plant growth-promoting genetic elements, these findings suggest that KK18 may serve as a promising candidate for agriculture.

**TABLE 1:** *Pseudomonas* sp. KK18 genome information

Parameters	Genomic features
Illumina statistics	
Total bp	2,591,768,228
Genomic coverage	357 ×
Read length	150 bp (paired-end)
High-quality reads	16.9344 M
High-quality reads Q30 bases	2.4032 G
ONT statistics	
Total bp	968,216,383
Genomic coverage	133 ×
High-quality reads N50 (bp)	23,765
Raw reads N50 (bp)	22,327
Genome	
Topology	Circular
Size (bp)	7,253,342
GC content (%)	61%
Completeness	100%
Contamination	0.36%
Contigs N50 (bp)	7,300,000
Contigs L50	1
Protein-coding genes	6,461
tRNA number	66
Highest 16S identity (EzbioCloud)	99.2%^*a*^
Highest ANI (OrthoANI)	98.74%^*a*^
Isolation date	2015-06-03
Collection site coordinates	−75.21136, 164.23553
Sample collection location	Box corer at a depth of 1,224 meters

^*a*^ Whole genome to *Pseudomonas* sp. NC02 (accession no. CP025624).

## Data Availability

This genome project has been registered at NCBI under accession number PRJNA1079050. Raw reads were deposited at the NCBI Sequence Read Archive (SRA) under PRJNA1079050, including Nanopore sequencing (SRX27649036) and Illumina sequencing (SRX27649035). The genome sequence has been deposited at GenBank under the accession number CP146605.1 with the assembly accessible through GCA_037094675.1. Other annotations have also been uploaded to Zenodo (10.5281/zenodo.14793495).
